# Vertical distribution of methanotrophic archaea in an iron-rich groundwater discharge zone

**DOI:** 10.1371/journal.pone.0319069

**Published:** 2025-02-24

**Authors:** Katsunori Yanagawa, Misaki Okabeppu, Sakiko Kikuchi, Fumito Shiraishi, Yumiko Nakajima, Akihiro Kano

**Affiliations:** 1 Faculty of Environmental Engineering, The University of Kitakyushu, Kitakyushu, Fukuoka, Japan; 2 Kochi Institute for Core Sample Research, Institute for Extra-cutting-edge Science and Technology Avant-garde Research (X-star), Japan Agency for Marine-Earth Science and Technology (JAMSTEC), Nankoku, Kochi, Japan; 3 Earth and Planetary Systems Science Program, Graduate School of Advanced Science and Engineering, Hiroshima University, Higashi-Hiroshima, Hiroshima, Japan; 4 Central Institute of Radioisotope Science and Safety Management, Kyushu University, Fukuoka, Japan; 5 Department of Earth and Planetary Science, Graduate School of Science, The University of Tokyo, Bunkyo-ku, Tokyo, Japan; Kobe University: Kobe Daigaku, JAPAN

## Abstract

Anaerobic oxidation of methane coupled to iron reduction (Fe-AOM) is a crucial process for methane removal in terrestrial environments. However, the occurrence of Fe-AOM in natural environments is rare, and the mechanisms behind the direct coupling of methane oxidation and iron reduction remain poorly understood. In this study, we investigated the environmental factors influencing the distribution of methanotrophic archaea in an iron-rich zone of a freshwater pond in Hiroshima Prefecture, Japan. High concentration of dissolved ferrous iron supplied by groundwater discharge led to considerable ferrihydrite precipitation. Pore water methane increased with sediment depth, while nitrate and sulfate concentrations were near detection limits throughout the sediment column. The coexistence of ferric iron and methane suggests the ongoing process of Fe-AOM. Tracer-based experiments using ^14^C showed potential Fe-AOM rates up to 110 pmol mL^-1^ day^-1^. Throughout the sediment core, except at the surface, PCR-based molecular ecological analyses of the 16S rRNA gene and functional genes for anaerobic oxidation of methane revealed abundant sequences belonging to the family “*Candidatus* Methanoperedenaceae”. These geochemical and microbiological findings suggest that Fe-AOM plays a key role in biogeochemical cycles of iron and methane, positioning this environment as a modern analogue of early Earth conditions.

## Introduction

Methane (CH_4_) is a major greenhouse gas, and its global warming potential is approximately 30 times greater than that of carbon dioxide (CO_2_) [1]. Global methane emissions from natural sources are estimated to be 218 Tg per year [2], with atmospheric methane concentrations increasing by 5.7 to 17.9 ppb annually over the past decade [3]. Methane is produced from various natural sources, including marine and freshwater sediments, wetlands, inland aquatic ecosystems, rice paddy soils, organic-rich landfills, and animal digestive systems. Among these emission sites, freshwater systems are considered as a major source of methane emissions to the atmosphere, contributing approximately 53% of the total global emissions [4].

Methane fluxes from natural environments are largely mitigated under anoxic conditions by a microbial process called anaerobic oxidation of methane (AOM), which operates via a reverse methanogenesis pathway. In marine sediments, AOM is typically coupled with sulfate (SO_4_^2-^) reduction [5,6]. Sulfate-AOM serves as the main sink for methane in marine environments, removing 70–90% of the methane released into the overlying seawater and atmosphere [7,8]. However, sulfate concentrations are considerably low in freshwater systems such as lakes and rivers [9], making sulfate-dependent processes less crucial in these systems [10,11]. Thermodynamic considerations based on free energy yield suggest that more favorable electron acceptors, such as nitrite (NO_2_^-^), nitrate (NO_3_^-^) [12], Mn(IV) [13], iron oxides [14–17], As(V) [18], and humic substances [19,20] could oxidize methane under anaerobic conditions. Among these, AOM coupled with metal oxides, such as Fe(III) reduction (Fe-AOM), has been identified as a key process for methane regulation in terrestrial [11], coastal [21,22], and marine environments [23].

Anaerobic methanotrophic archaea belonging to ANME-1, -2, and -3 are responsible for sulfate-AOM in various marine environments [5]. By contrast, Fe-AOM in freshwater environments is primarily mediated by members of the family “*Candidatus* Methanoperedenaceae” [15,24–26]. This group of archaea belongs to the ANME-2d cluster, previously described as AOM-associated archaea (AAA) [10], and consists solely of uncultured representatives from terrestrial habitats, although some marine sequences have also been identified [27]. Unlike in previous studies reporting that ANME show syntrophic associations with bacterial partners, *Ca.* Methanoperedenaceae can perform Fe-AOM without bacterial partners [15] and can utilize various electron acceptors other than Fe(III), such as NO_3_^-^ [12], SO_4_^2-^ [26,28], Mn(IV) [13], Cr(VI) [29], and humic substances [19].

Although many studies have been conducted on Fe-AOM, most of them have focused on enrichment cultures for archaeal methanotrophs. Thus, the role of *Ca.* Methanoperedenaceae in natural methane mitigation remains unclear. Consequently, the key factors controlling Fe-AOM and the environmental distribution of these methanotrophs also remain relatively unexplored. The occurrence of Fe-AOM in natural environments needs to be further explored to determine which forms of iron oxide act as electron acceptors for Fe-AOM [30]. In this study, we investigated freshwater sediments from a groundwater discharge zone in Budo Pond, Hiroshima Prefecture, Japan. Owing to the supply of Fe^2 + ^-rich underground fluids, ferruginous sediments primarily composed of biogenic ferrihydrite are established at the mouth of the groundwater discharge in Budo Pond [31–33]. These deposits were observed throughout the year within an area several meters from the discharge point. Deep sediments are characterized by anaerobic conditions with abundant methane produced by methanogenic archaea [31]. Therefore, the study site provides an ideal habitat for microorganisms involved in Fe-AOM. In the present study, based on a detailed vertical analysis conducted at centimeter-scale intervals in iron-rich habitats using an integrated approach, including geochemistry, stable isotope analysis, radiotracer experiments, microbial abundance quantification, and molecular ecology, we examined the distribution and favorable niches of *Ca*. Methanoperedenaceae, the key microbes controlling methane reduction via Fe-AOM.

## Materials and methods

### Sample collection and processing

Budo Pond is located on the campus of Hiroshima University, Japan (34°24.060 N, 132°42.790 E) (S1 Fig). Sample collection was conducted with the approval of Hiroshima University. Before sampling the sediment core, the pH, temperature, dissolved oxygen (DO), and redox potential (Eh) of the surface water were measured on-site using a portable sensor (D-75, Horiba, Kyoto, Japan) equipped with individual electrodes. In August 2016, a sediment core was collected at 20 cm from the groundwater discharge point using an acrylic tube with an inner diameter of 6 cm. The core was immediately sectioned into 1–4 cm-thick slices for geochemical and molecular biological analyses using sterile spatulas. Samples for DNA analysis were stored at −80 °C in 1.5 mL microcentrifuge tubes. A portion of the sediment was placed in 50 mL conical tube and centrifuged to extract pore water. The supernatant was filtered through a 0.45 μm pore-size cellulose acetate filter and stored until further analysis. Another 1 mL portion of the sediment was placed in a 15 mL conical tube, fixed overnight at 4 °C with a 3.7% formaldehyde solution, and stored at −80 °C until microscopic examination. For AOM activity measurement, 5 mL of sediment samples were stored anaerobically at 4 °C in 30 mL glass vials, with the headspace replaced by argon gas.

### Water geochemistry

The major cations (Na^ + ^, Mg^2 + ^, K^ + ^, and Ca^2+^) and anions (Cl^-^, NO_3_^-^, and SO_4_^2-^) in the pore water were measured using ion chromatography (IC; Jasco Gulliver system, Jasco International Co., Ltd, Tokyo, Japan). The concentration of Mn^2 +^ was evaluated using atomic absorption spectroscopy (AA-6200; Shimadzu, Kyoto, Japan). The concentrations of dissolved Fe^2 +^ and hydrogen sulfide were measured spectrophotometrically using the ferrozine [34] and methylene blue [35] methods, respectively. Dissolved inorganic carbon (DIC) and methane were measured using the headspace gas method, as described previously [36], using a gas chromatograph (GC; Agilent 7820A) equipped with a thermal conductivity detector. The stable carbon isotopic compositions of DIC (δ^13^C_DIC_) were measured using a conventional isotopic ratio mass spectrometer (IRMS; Thermo Finnigan Delta Plus XP) connected to a Flash EA 1112 Automatic Elemental Analyzer via a ConFlo III interface as described previously [37]. Total organic carbon (TOC) concentrations were determined using an elemental analyzer (CHN CORDER MT-6, Yanako). The δ^13^C_TOC_ values were measured using an isotope ratio mass spectrometer (Isoprime VisION, Elementar) coupled with an elemental analyzer (vario PYRO cube, Elementar).

### AOM rates

The potential of AOM was assessed using radioisotope tracer incubation experiments, as described previously [38,39]. In brief, 5 cm^3^ of sediment samples were incubated at 20 °C for five days in darkness under a headspace gas filled with 200 kPa of methane and 1 MBq of ^14^C-labeled methane (American Radiolabeled Chemicals, Inc., St. Louis, MO, USA). Two autoclaved samples were used as negative controls. To terminate the microbial activity and facilitate the release of CO_2_ into the headspace, the samples were acidified with 1 mL of 6 N HCl. The radioactivity of a portion of reaction products (i.e., ^14^CO_2_) in the headspace was determined using a gas chromatograph (Shimadzu GC8A, Shimadzu, Kyoto, Japan) equipped with a high-sensitivity radioactivity detector (RAGA Star, Raytest, Straubenhart, Germany). Potential AOM activities were calculated using following equations:


AOMrate=k×C,

$$Turnoverrate\left(k\right)=F\times \frac{a{-}^{14}CO_{2}}{a{-}^{14}CH_{4}}\times \frac{1}{t},$$

where C is the *in situ* methane concentration, F is the isotope fraction factor for AOM (1.06 [40]), a-^14^CO_2_ is the radioactivity of the produced ^14^CO_2_, a-^14^CH_4_ is the radioactivity of the injected ^14^CH_4_, and t is the incubation time (5 days). In this equation, the AOM rate is expressed as pmol CH_4_ oxidized per mL sediment per day.

### Prokaryotic 16S rRNA gene phylotype composition analysis

Microbial DNA was extracted directly from 0.2 g of sediment using the DNeasy PowerSoil Kit (Qiagen, Hilden, Germany). Microbial cells were mechanically crushed for 10 min using a μT-01 bead crusher (TAITEC, Koshigaya, Japan) as described previously [41]. The extracted DNA samples were stored at −80 °C for further analyses. Universal primers 515F and 806R [42] were used to amplify the hypervariable V4 region of the prokaryotic 16S rRNA gene. Amplification was performed using MightyAmp DNA Polymerase Ver.3 (Takara Bio) and Biometra TAdvanced 96 SG (Biometra, Göttingen, Germany). The thermal cycling conditions comprised an initial denaturation step at 98 °C for 2 min, followed by 40 cycles of denaturation at 98 °C for 30 s, annealing at 55 °C for 30 s, extension at 68 °C for 30 s, and a final extension at 68 °C for 5 min. An equimolar mixture of all PCR products was sent to FASMAC Co., Ltd. for 2 ×  300 bp paired-end sequencing on the Illumina MiSeq platform using the Illumina MiSeq Reagent Kit v3. Downstream analysis of sequence reads was performed using QIIME 2 2020.8 [43], including DADA2 [44]. The 16S rRNA gene amplicon sequences were aligned with MAFFT [45] and used to construct a phylogenetic tree using FastTree [46]. Taxonomic identification of the representative sequences was performed using the SILVA 138 database (silva-138-99-nb-classifier). Alpha and beta diversity analyses were performed using core-metrics-phylogenetic plugin in QIIME 2. Beta diversity distances were determined based on Bray-Curtis distances. Raw sequence reads were deposited in the Sequence Read Archive (SRA) under the accession number DRA016493.

### Microbial cell and gene abundance

Approximately 0.2 g of the sediment sample was preserved in a 3% formaldehyde solution for 2 h at room temperature. The fixed microbial cells were filtered onto 0.2 μm-pore-size polycarbonate membranes (Isopore Membrane, Merck Millipore, USA). To preserve the integrity of cell aggregates, no dispersing treatments, such as sonication, were applied prior to filtration. The cells were stained with 250 × SYBR Green I, and counted in triplicate, as described previously [47]. Microscopic images were captured at magnifications ranging from 400 × to 1000 × using an Eclipse 80i fluorescence microscope (Nikon, Tokyo, Japan) equipped with B-2A longpass filter cubes.

Quantification of total prokaryotic and archaeal 16S rRNA gene abundance was performed via quantitative PCR (qPCR) using universal and archaeal primer-probe sets [48] and an innuMIX qPCR MasterMix Probe and a real-time PCR system qTOWER^3^ G touch (Analytik Jena AG, Germany). The thermal cycling conditions were as follows: 50 cycles of denaturation at 98 °C for 10 s, annealing at 50 °C (for the universal 16S rRNA gene) or 52 °C (for the archaeal 16S rRNA gene) for 45 s and an extension at 72 °C for 30 s. *mcrA* gene abundance was also determined using a specific primer set [49] and MightyAmp for Real-Time PCR (TaKaRa Bio, Inc., Otsu, Japan). The amplification conditions comprised 40 cycles of denaturation for 40 s at 94 °C, annealing at 52 °C for 30 s, and extension at 68 °C for 60 s. All qPCR assays were performed in triplicate. Details of the qPCR experiments are provided in S1 Table.

### Clone library construction of *mcrA* genes

The *mcrA* gene fragments were amplified via PCR using MightyAmp DNA Polymerase Ver.3 (TaKaRa Bio, Inc., Otsu, Japan) and the specific primer set used for the qPCR analysis [49]. The amplified PCR products were purified, cloned, and sequenced as described previously [9]. The taxonomic affiliations of the *mcrA* gene sequences were determined based on neighboring reference sequences in phylogenetic tree, which was constructed using a custom-curated *mcrA* dataset with the ARB software package [50]. The *mcrA* gene sequences reported in this study have been deposited in the DDBJ/EMBL/GenBank databases under accession numbers LC770274–LC770306.

## Results and discussion

### Geochemical features of Budo Pond sediment

**Fig 1:**
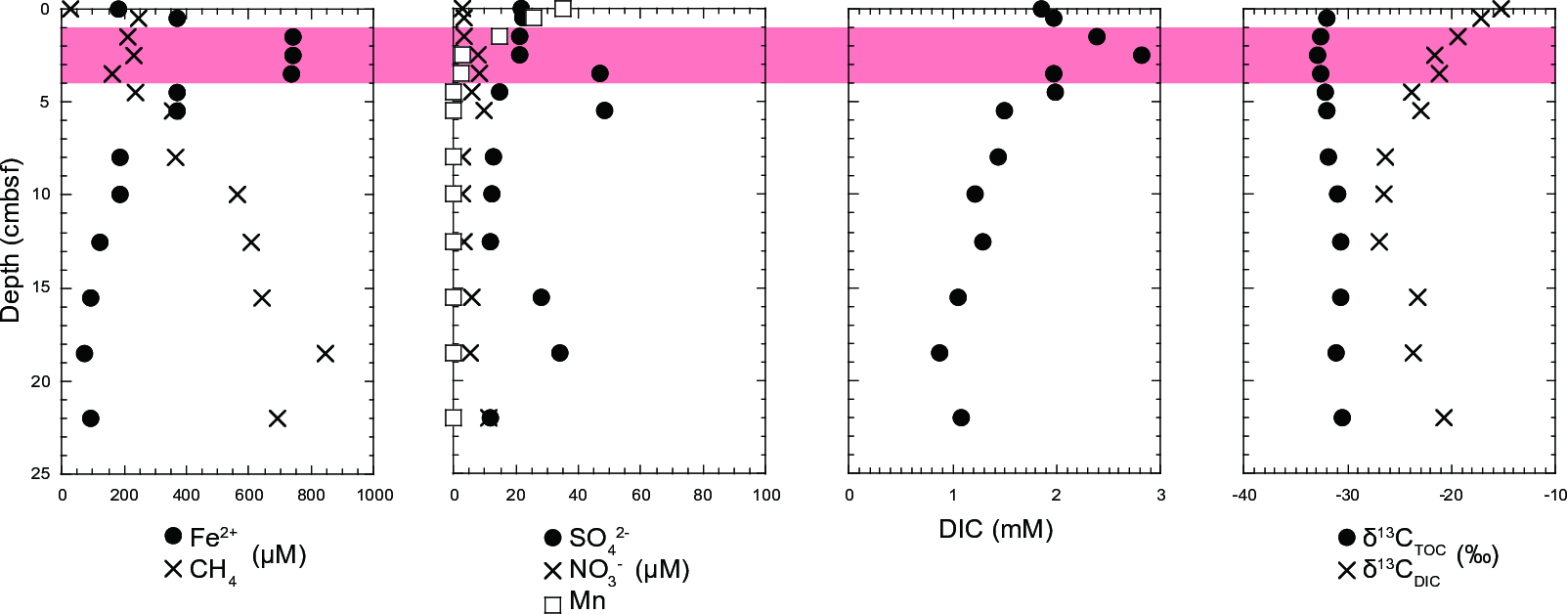
Depth profiles of pore water geochemistry in the Budo Pond sediment. The red-shaded layers indicate the depth range of high iron concentration.

The temperature of the discharged groundwater was 17.3 °C, slightly higher than the average annual temperature of 16 °C [31]. The pH was slightly acidic at 6.3, with dissolved oxygen concentration of 0.75 mg/L (23.4 µ M) and an Eh value of −23 mV. CH_4_ concentrations were significantly lower in the shallow sediments but higher in the deeper sediments, reaching up to 850 μM at a depth of 18.5 cm (Fig 1 and S2 Table). Dissolved iron concentration (Fe^2+^) was 740 μM at 1.5–3.5 cm depths but decreased to 70 μM at 18.5 cm from the surface. The positive iron peak corresponded to the negative methane peak. The concentrations of SO_4_^2-^, NO_3_^-^, and Mn were less than 48.5, 11.4, and 34.9 µ M, respectively. No considerable changes in the vertical profiles of SO_4_^2-^, NO_3_^-^, and Mn concentrations were observed in relation to the CH_4_ profile. Furthermore, sulfide concentration was below the detection limit of 0.5 µ M at all depths. The depth profile of dissolved inorganic carbon (DIC), one of the major end-products of AOM, mirrored the Fe^2 +^ profile, increasing from 1.8 mM (0 cm depth) to 2.8 mM (2.5 cm) before decreasing to approximately 1 mM at 15.5 cm. Calcium (Ca^2+^) concentrations exceeded 0.2 mM in the upper sediment but were reduced by half in the deepest part (S2 Fig), consistent with the notion that Ca^2 +^ are used to form calcium carbonates under high pH condition. In marine environments, high bicarbonate concentrations lead to the precipitation of calcium carbonate [8], which is often interpreted as a fossil signature of AOM [51,52]. The vertical distributions of other ions such as Na^ + ^, K^ + ^, and Cl^-^ showed no notable variation. These physicochemical characteristics suggest that iron reduction may play a key role in shaping the CH_4_ profile. They are not only consistent with those previously reported at this discharge point [31–33] but also similar to findings from other iron-rich freshwater sediments where AOM occurs [28].

The stable carbon isotope compositions of the TOC and DIC were also determined. The δ^13^C_DIC_ value of the surface pore water was −15.2‰ VPDB (Vienna Pee Dee Belemnite), likely affected by inorganic carbon in the pond water and the atmosphere. It significantly decreased to −23.9‰ VPDB at 4.5 cm depth (Fig 1). This steep decline could be attributed to the involvement of DIC derived from the oxidation of methane and/or organic materials in the sediment. According to a previous study [31], δ^13^C_methane_ was −68.8‰ VPDB. TOC concentrations in the sediments were relatively high (0.4–4 wt%) (S2 Fig). The δ^13^C_TOC_ values ranged from −32.9‰ to −30.5‰ VPDB (Fig 1), which are slightly lower than the values typically observed in freshwater sediments. The lowest value was observed at a depth of 2.5 cm, which was attributed to the influence of methanotroph-derived organic matter, as this depth corresponds to the potential Fe-AOM zone.

### AOM activity

**Fig 2:**
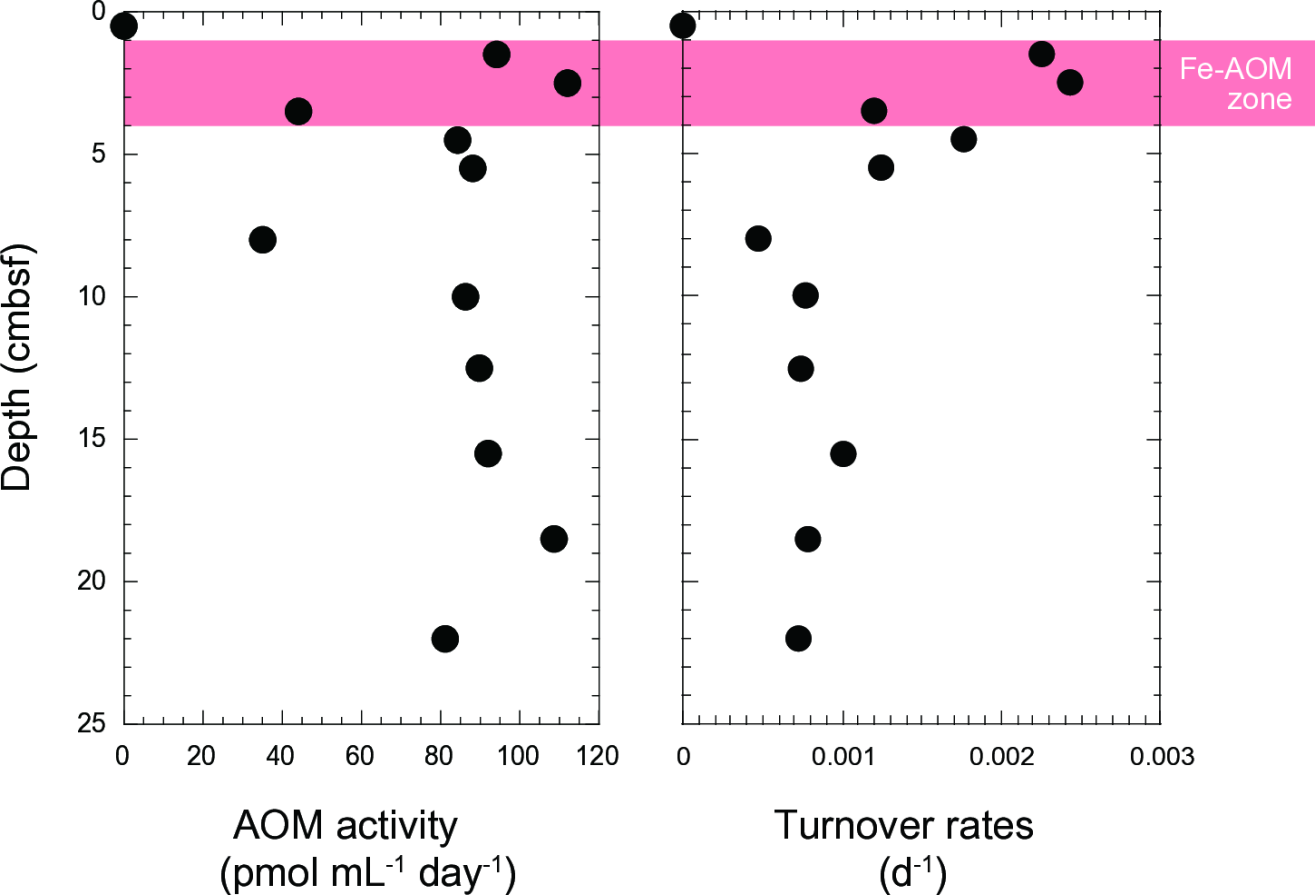
Depth profiles of potential AOM activity and turnover rates.

We explored the potential role of Fe(III) as a terminal electron acceptor in AOM using the sediment from Budo Pond. Experiments utilizing radiolabeled tracers revealed the maximum AOM rate of 110 pmol mL^-1^ day^-1^ (Fig 2). Given the high amounts of ferrihydrite in the sediments, Fe(III) was likely the primary electron acceptor and played a crucial role in methane removal. The AOM rates in this study were measured at 20°C, which is slightly higher than the in situ water temperature of 17.3°C. While this may have led to overestimation of the AOM rate, the values remain comparable to those reported in iron-amended incubation experiments conducted in terrestrial subsurface environments [53]. Although these rates were significantly lower than those reported for enrichment culture samples obtained from surface environments [15], this discrepancy is likely due to the differences in sample types (enrichment culture vs. natural environment) as well as the abundance and activity of anaerobic methanotrophs. The biological methane production rate in deeper sediments has not been investigated; however, the turnover rate observed in tracer experiments suggests that methane in the studied core could be depleted through AOM within a few years (Fig 2). This finding underscores the importance of this process in regulating methane concentrations within freshwater systems.

### 16S rRNA gene-based microbial community structure

**Fig 3:**
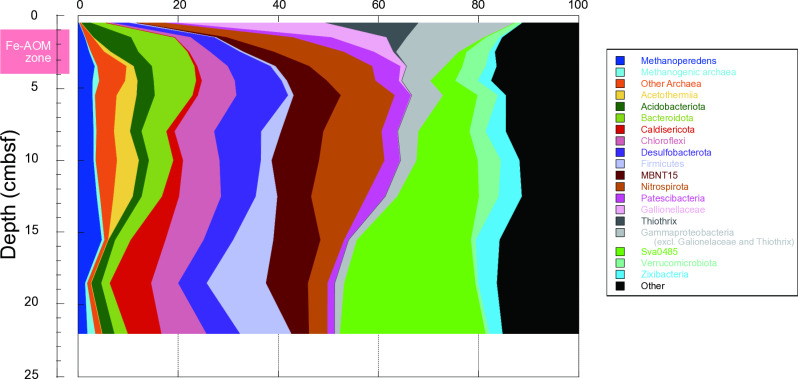
Taxonomic compositions of 16S rRNA gene sequences in the sediments. The width of the bars along the vertical axis corresponds to the actual range of each core section.

To identify the methanotrophic archaea potentially responsible for Fe-AOM at our study site, we analyzed the microbial community compositions of 12 sediment layers through amplicon sequencing targeting the V4 hypervariable region of the 16S rRNA gene fragments. A total of 877,716 quality-filtered sequences were obtained and used for further analysis (Fig 3 and S3 Table).

Members of genus *Ca.* Methanoperedens, within the class Methanosarcinia of the phylum Halobacterota, were detected in the 16S rRNA gene phylotype. They represent one of the most abundant archaeal populations at the genus level, comprising up to 4.7% of the total community composition. At the top surface, where oxic and low methane conditions prevailed, they accounted for only 0.2% of the community. No sequences of other archaeal methanotrophs were detected at any depth in the sediment core from Budo Pond. In contrast, methanogenic archaea constituted less than 0.4% of the microbial community throughout the sediment.

The vertical profile of the relative abundance of the 16S rRNA gene phylotype composition revealed that members belonging to the family Gallionellaceae predominated in the surface sediment, comprising up to 29.6% of all phylotypes. Most were classified within the genera Gallionella and Sideroxydans, indicating the dominance of Fe(II)-oxidizing bacteria, which is similar to the surface environment of many iron-rich freshwater systems [54]. Additionally, members of the genus Thiothrix in the family Thiotrichaceae accounted for 18.6% of the total community composition in the surface sediment. They are known for sulfur oxidization [55] despite the absence of sulfide in the pore water throughout the core.

In the middle core layers, the major phylotypes changed to the phyla Bacteroidetes, Chloroflexi, Desulfobacterota, Nitrospirota, MBNT15, Nitrospirota, Verrucomicrobiota, and Zixibacteria. Among the Desulfobacterota, members of the genus Syntrophus, known for their syntrophic lifestyle [56], were prominent and accounted for 3.4–4.5% at depths of 3.5–12.5 cm. The candidate phylum MBNT15 was abundant at depths of 2.5–18.5 cm. Most members perform dissimilatory iron reduction according to metagenomic analyses [57]. Members of the Verrucomicrobiota included the genus “*Candidatus* Omnitrophus,” which accounted for 3.4–4% at depths of 10–12.5 cm. A previous report suggested that genetic potential of Omnitrophus is associated with magnetosome biosynthesis, sulfur oxidation, and carbon fixation [58]. Members of the phylum Zixibacteria were abundant in the deepest layers. They are considered potential participants in iron cycling within the continental subsurface [59]. Notably, previous research has specifically highlighted the iron-reducing metabolic capabilities of Zixibacteria in coastal sediments [60].

At the bottom of the core, community members shifted and the bacterial phyla Caldisericota, Firmicutes, and Sva0485 became more prominent. Members of Caldisericota were particularly dominant at a depth of 18.5 cm, comprising 8.3% of the overall community. These organisms are heterotrophs [61] and thrive on oxidized sulfur intermediates. Although these sulfur compounds could be provided by sulfur-oxidizing bacteria or by the oxidation of sulfides by ferric iron (Fe^3+^) within the sediments, the concentrations of sulfides in the pore water were below the detection limits, suggesting a small-scale sulfur cycle. Below a depth of 18.5 cm, members of the phylum Firmicutes comprised more than 10.3% of the community. Notably, the uncultured Class D8A-2 accounted for more than 92% of Firmicutes, representing the majority. They possess the potential for syntrophic propionate oxidation [62]. The most notable increase in depth was observed in members of Sva0485, which accounted for 29.1% of the community at the deepest point of 22 cm. These have been described as most likely sulfate reducers or sulfur oxidizers, depending on the redox conditions [63]. Additionally, several genes related to the iron cycle have also been identified from the Sva0485 MAGs. Potential Fe(III)-reducing bacterial lineages commonly found in natural environments, such as Geobacteraceae [64], were detected as minor populations, each constituting less than 0.1% of the community.

The alpha diversity metrics including the number of observed features, the Shannon index, Pielou’s evenness, and Faith’s PD are shown in S3 Table and S3 Fig. These alpha diversity indices varied significantly with depth, showing a notable decreasing trend with greater depths. The beta diversity analysis suggested that the microbial community composition changed continuously with depth. (S4 Fig).

### Quantitative analysis of microbial populations

**Fig 4:**
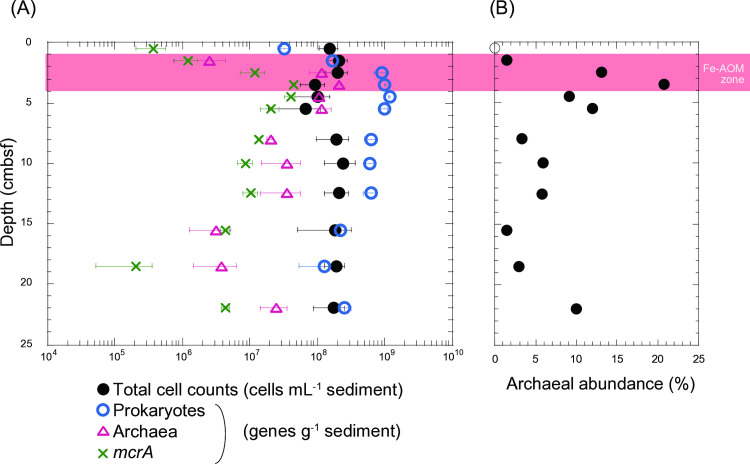
Microbial biomass in the Budo Pond sediment. (A) Total cell counts (black circles) and the 16S rRNA gene numbers of prokaryotes (blue circles), archaea (red triangles), and *mcrA* (green crosses) quantified by qPCR. (B) The relative archaeal abundances determined by the ratio of the number of archaeal 16S rRNA genes to that of total prokaryotic 16S rRNA genes. Open circles on the y-axes denote analyses below the detection limit.

Fluorescence microscopy revealed microbial cell concentrations ranging from 6.7 × 10^7^ to 2.5 × 10^8^ cells per mL of the sediment (Fig 4). All the observed cells appeared as individual entities (S5 Fig), indicating the absence of the ANME consortia with specific bacterial partners. The qPCR analysis showed similar levels of total prokaryotic 16S rRNA genes, ranging from 3.3 × 10^7^ to 1.2 × 10^9^ genes per gram of sediment. This supports the idea that most of cells observed under the microscope are prokaryotes. Archaeal 16S rRNA gene profiles followed a similar trend but were one to two orders of magnitude lower than those of prokaryotes, with values ranging from 2.6 × 10^6^ to 2.1 × 10^8^ genes per gram of sediment.

**Fig 5:**
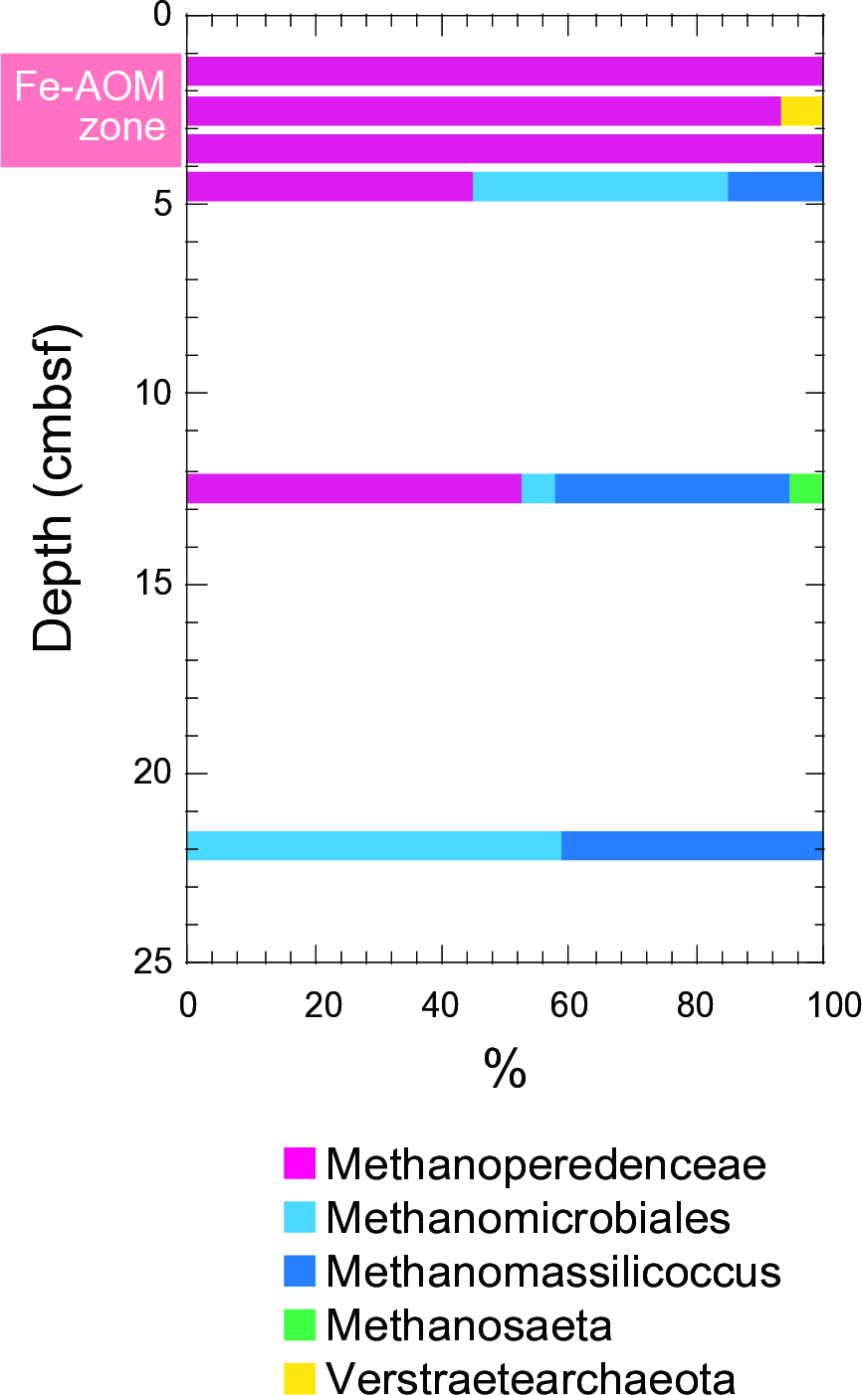
Phylogenetic affiliation of *mcrA* genes.

The abundance of *mcrA* gene, which is a key functional gene for AOM or methanogenesis, followed a similar pattern but was relatively lower than that of the archaeal 16S rRNA genes (Fig 4). The highest *mcrA* concentration was detected in shallow sediment (3.5 cm) with 4.5 ×  10^7^ copies per gram of sediment. The relative composition of the *mcrA* phylotypes was determined through sequencing of the *mcrA* genes amplified (Fig 5). Phylogenetically, *mcrA* was categorized into several groups, including Methanoperedenaceae, Methanomicrobiales, Methanomassilicoccus, Methanosaeta, and Verstraetearchaeota. At depths of approximately 1.5 to 3.5 cm, over 93% of the *mcrA* gene amplicons were associated with the Methanoperedenaceae.

### Factors controlling Fe-AOM

The significant increase in pore water Fe^2 +^ concentrations between 1.5 and 3.5 cm depths suggests that Fe(III) reduction occurred in the specific anoxic layer. This coincided with the positive peaks of DIC and Methanoperedenaceae *mcrA* gene abundance as well as negative methane trends, indicating that methane removal was coupled with dissimilatory iron reduction and simultaneous CO_2_ generation. Additionally, the δ^13^C_TOC_ value exhibited a slight peak and dropped down to −32.9 ‰ VPDB at 2.5 cm. These relatively low values measured in the AOM zone imply the active accumulation of methane-derived carbon in organic materials. The correlation between the gene abundance of Methanoperedenaceae *mcrA* and AOM rate further supports the role of Methanoperedenaceae in methane oxidation. This idea is corroborated by the fact that Methanoperedens is dominant in environments where Fe- AOM occurs [15,24,53]. Although the possibility of the involvement of NO_3_^-^, SO_4_^2-^, and Mn(IV) reduction cannot be entirely excluded, their concentrations did not provide a significant portion of the AOM in Budo Pond. These values were much lower than the Fe^2 +^ concentrations. NO_3_^-^ and SO_4_^2-^concentrations showed slight fluctuations, with no correlation to methane. Therefore, Fe(III) appears to be a thermodynamically favorable electron acceptor for AOM in Fe(III)-rich sediments. The exact mechanism of Fe(III) utilization by Methanoperedenaceae is not yet fully understood. In this study, no cell aggregates were observed using fluorescence microscopy, suggesting that there was no physical interaction between the Methanoperedenaceae archaeons and bacterial partners (S5 Fig). Some members of Methanoperedenaceae perform Fe-AOM via direct electron transfer without bacterial partners involving multi-heme c-type cytochromes [15,24]. This process is facilitated by an extracellular electron transport mechanism wherein electrons generated during methane oxidation are relayed through multi-heme c-type cytochromes to the terminal electron acceptors, such as ferrihydrite. Furthermore, it has been suggested that electrons might pass on to dissimilatory metal-reducing bacteria, such as *Shewanella oneidensis* [65].

Despite the low activity of our samples, the radiotracer-based incubation experiments revealed relatively high AOM rates at a depth of 2.5 cm. This aligned with the qPCR results, which showed a high abundance of Methanoperedenaceae *mcrA* at this depth. The availability of electron acceptors suggests that Fe(III) is a key factor controlling Fe-AOM activity in surface sediments. Fe(III)-containing minerals might have formed from biotic or abiotic Fe^2 +^ oxidation near the sediment surface. The 16S rRNA gene amplicon analysis suggested that members of Gallionellaceae contributed to Fe(II) oxidation in surface sediments, as shown in a previous study at this site [32]. The biogenic Fe(III) materials produced, known as stalks and sheaths, are composed of structurally less ordered and complex ferrihydrites and are characterized by large surface areas [66]. These less crystalline ferrihydrites are more susceptible to reduction by dissimilatory Fe(III)-reducing bacteria [67]. These biologically derived ferrihydrite-like materials were also abundant at the bottom of the core. In the deeper layers, Fe(III) is dissimilatorily reduced to Fe(II) through microbial processes, leading to the formation of secondary iron minerals, such as goethite and siderite [68–70]. In Budo Pond, both ferrihydrite and goethite coexist in deep sediments; however, the surface of ferrihydrite in deeper sediments is partially covered with goethite [31]. These findings suggest that while surface sediments provide specific physical and chemical conditions suitable for Fe-AOM, unfavorable conditions exist in deeper sediments. Indeed, the vertical profile of the *mcrA* gene and the Fe²⁺ concentration in pore water indicated that Fe-AOM is not occurring throughout the core but is limited to surface sediments at depths of 1.5–3.5 cm (Figs 1 and 4). We hypothesized that the encrustation of ferrihydrite surfaces by goethite is a likely reason why Methanoperedenaceae cannot access ferrihydrite, thereby reducing the potential activity of Fe-AOM.

### Environmental significance of Fe-AOM in iron-rich sediments

Several previous studies have recognized Fe-AOM as an important methane removal process in various iron-rich environments based on geochemical evidence [16,21,22,71–74]. However, the microorganisms responsible for the metal-dependent AOM remain largely unknown. This study demonstrated that in freshwater sediments where methane and iron oxides coexist and potential electron acceptors, such as SO_4_^2-^, are almost absent, Fe-AOM serves as a significant methane sink, with Methanoperedenaceae identified as the primary microorganisms involved in this process. This allowed for a discussion on the importance of specific forms of iron and the quantitative distribution of the microorganisms involved. Consistent with our findings, Methanoperedenaceae have been reported as prominent candidates for Fe-AOM processes in low-sulfate, iron-rich environments such as the anoxic water column of Kabuno Bay [75], freshwater sediments of Lake Ørn [28,76] and Lake Cadagno [10], freshwater wetland [77], alpine wetlands [25], and deep groundwater [53]. These environments are generally characterized by high sedimentation rates or substantial iron oxide inputs from rock weathering or anthropogenic sources, providing the necessary electron acceptors for metal-dependent AOM. The geochemical features of Budo Pond include low sulfate levels and anoxic iron-rich conditions with Fe²⁺ concentrations reaching 740 μM at a depth of just a few centimeters. These geochemical conditions are thought to serve as modern analogs of the Archaean oceans, which had vastly different ocean-atmosphere systems compared to those existing today. Before the Great Oxidation Event (GOE), the atmosphere had low oxygen levels, and seawater contained low sulfate levels, generally being anoxic and ferruginous [78–80]. Due to the GOE, iron-rich sediments were widely deposited. Fe-AOM processes may have played a crucial role in methane removal in the early Earth’s oceans by linking carbon and iron cycles, preventing methane from diffusing into the overlying water or atmosphere, and significantly influencing the climatic conditions of early Earth.

## Conclusions

AOM occurred at the groundwater discharge point of Budo Pond. The potential rate was determined using radiotracer-based activity measurements, which revealed the highest rate at a depth of 2.5 cm in the upper sediment. Several geochemical features were observed in the shallow zone: (1) methane depletion with Fe^2 +^ enrichment, (2) levels of other electron acceptors several orders of magnitude lower, (3) production of isotopically light DIC, and (4) a distinct peak of isotopically light TOC. Molecular biology analyses targeting the 16S rRNA and *mcrA* genes revealed the dominance of the anaerobic methanotrophs of Methanoperedenaceae. All lines of evidence suggest that methane removal is primarily coupled with iron reduction in this ferruginous freshwater system, with Methanoperedenaceae members identified as the microorganisms responsible for the observed Fe-AOM. The fresh supply of reactive iron oxides likely stimulates Fe-AOM in the surface sediments of Budo Pond, making it an ideal site for further studies on the role and regulation of AOM in natural environments with elevated iron concentrations.

## Supporting information

S1 Table Details of qPCR experiments conducted in this study. (PDF)

S2 Table Summary of geochemical and microbiological raw data used in this study. (PDF)

S3 Table Summary of the 16S rRNA gene amplicon libraries. (PDF)

S1 Fig Location of sampling sites. Overview of the groundwater discharge point of Budo Pond.
(PDF)

S2 Fig Depth profiles of pore water Na, K, Cl, Mg, Ca, TOC, and the oxygen isotopic composition of water. The red-shaded layers represent the possible depth ranges of active Fe-AOM, as determined in Fig 1.
(PDF)

S3 Fig Rarefaction curves for the 16S rRNA gene amplicon libraries of the sediment. (PDF)

S4 Fig Beta-diversity visualized using principal coordinate analysis (PCoA). The two principal coordinate axes explain 75.83% of variation.
(PDF)

S5 Fig Fluorescence microscopic image of microbial cells from the Budo Pond sediment. Sediment samples at depths of 1.5 cm (A) and 4.5 cm (B) were selected as examples to show cell morphology.
(PDF)
