# Supplementary material for: Vertical distribution of methanotrophic archaea in an iron-rich groundwater discharge zone
Source: PLoS One. 2025 Feb 24;20(2):e0319069. doi: 10.1371/journal.pone.0319069 (PMC11849818; doi:10.1371/journal.pone.0319069)
Supplement: S1 Table — (PDF) [file pone.0319069.s001.pdf]

S1 Table.Details of qPCR experiments conducted in this study

	Prokaryotic Universal 16S rRNA gene	Archaeal 16S rRNA gene	*mcrA*
Forward primer	Uni340F (5'-CCTACGGGRBGCASCAG-3')	Arch349F (5'-GYGCASCAGKCGMGAAW-3')	ME3MF (5'-ATGTCNGGTGGHGTMGGSTTYAC-3')
Reverse primer	Uni806R (5'-GGACTACNNGGGTATCTAAT-3')	Arch806R (5'-GGACTACVSGGGTATCTAAT-3')	ME2r' (5'-TCATBGCRTAGTTDGGRTAGT-3')
Taqman Probe	Uni516F (5'-TGYCAGCMGCCGCGGTAAHACVNRS-3')	Arch516F (5'-TGYCAGCCGCCGCGGTAAHACCVGC-3')	—
Standard curve range	4.5×10^2 – 4.5×10^7 copies/μL	1.1×10^2 – 1.1×10^7 copies/μL	9.6×10^2 – 9.6×10^7 copies/μL
R^2 value	0.997	0.995	0.998
PCR efficiency (%)	0.81	0.74	0.83
Quantification method	Absolute quantification	Absolute quantification	Absolute quantification
Threshold value	7.2	7.3	1.3
Reaction volume	20 μL	20 μL	20 μL
Reaction mixture composition			
Master mix	innuMIX qPCR MasterMix Probe	innuMIX qPCR MasterMix Probe	MightyAmp for Real-Time PCR
Primer concentration	0.2 μM	0.2 μM	0.2 μM
Template DNA	1 μL	1 μL	1 μL
Cycling conditions	50 cycles of 98°C for 10 s, 50°C for 45 s, and 72°C for 30 s	50 cycles of 98°C for 10 s, 52°C for 45 s, and 72°C for 30 s	40 cycles of 94°C for 40 s, 52°C for 30 s, and 68°C for 60 s
qPCR machine	qTOWER^3 G touch	qTOWER^3 G touch	qTOWER^3 G touch
Reference	48	48	49
