# Supplementary material for: Vertical distribution of methanotrophic archaea in an iron-rich groundwater discharge zone
Source: PLoS One. 2025 Feb 24;20(2):e0319069. doi: 10.1371/journal.pone.0319069 (PMC11849818; doi:10.1371/journal.pone.0319069)
Supplement: S2 Table — (PDF) [file pone.0319069.s002.pdf]

S2 Table. Summary of geochemical and microbiological raw data used in this study.

Description	Depth (cm)	Na (μM)	Mg (μM)	K (μM)	Ca (μM)	Cl (μM)	SO_4 (μM)	NO_3 (μM)	Mn (μM)	Fe^{2+} (μM)	H_2S (μM)	CH_4 (μM)	TOC (%)	$\delta^{13}C_{TOC}$ (‰)	DIC (mM)	$\delta^{13}C_{DIC}$ (‰)	$\delta^{18}O$ (‰)	AOM activity (pmol mL^{-1} day^{-1})	Turnover rates (d^{-1})	Cell counts (cells mL^{-1} sediment)	Prokaryotic 16S rRNA gene (genes g^{-1} sediment)	Archaeal 16S rRNA gene (genes g^{-1} sediment)	*mcrA* gene (genes g^{-1} sediment)
Surface water	0	680	160	97	270	240	21	2.7	34	180	ND*	29			1.8	-15.2	-8.3						
0-1 cm	0.5	600	160	75	250	170	22	3.5	25	360	ND	240	3.4	-32.1	2.0	-17.2	-8.5	0	0	1.6.E+08	3.3.E+07	ND	3.8.E+05
1-2 cm	1.5	680	150	95	210	220	21	3.3	14	740	ND	200	2.6	-32.7	2.4	-19.3	-8.3	94	0.0022	2.1.E+08	1.7.E+08	2.6.E+06	1.2.E+06
2-3 cm	2.5	640	130	90	200	280	21	7.9	3.0	740	ND	220	2.6	-32.9	2.8	-21.6	-8.2	110	0.0024	2.1.E+08	9.1.E+08	1.2.E+08	1.2.E+07
3-4 cm	3.5	930	120	120	190	210	46	8.5	2.2	730	ND	160	2.2	-32.6	2.0	-21.2	-8.2	44	0.0011	9.4.E+07	1.0.E+09	2.1.E+08	4.5.E+07
4-5 cm	4.5	750	150	120	190	200	14	5.7	ND	370	ND	230	2.5	-32.2	2.0	-23.8	-8.2	84	0.0017	1.0.E+08	1.2.E+09	1.1.E+08	4.0.E+07
5-7 cm	6	1100	100	120	140	250	48	9.5	ND	360	ND	350	2.6	-32.0	1.5	-23.0	-8.3	88	0.0012	6.7.E+07	9.8.E+08	1.2.E+08	2.1.E+07
7-9 cm	8	620	130	110	140	180	12	2.7	ND	180	ND	360	2.4	-31.9	1.4	-26.4	-8.3	35	0.00047	1.9.E+08	6.3.E+08	2.1.E+07	1.4.E+07
9-11 cm	10	650	120	120	140	180	12	2.7	ND	180	ND	560	2.4	-31.0	1.2	-26.5	-8.3	86	0.00076	2.5.E+08	6.0.E+08	3.6.E+07	8.9.E+06
11-14 cm	12.5	700	110	120	130	180	11	3.3	ND	120	ND	600	2.0	-30.7	1.3	-27.0		89	0.00074	2.1.E+08	6.3.E+08	3.6.E+07	1.0.E+07
14-17 cm	15.5	830	100	120	97	190	28	5.7	ND	91	ND	640	0.74	-30.7	1.1	-23.3		91	0.00099	1.9.E+08	2.2.E+08	3.3.E+06	4.4.E+06
17-20 cm	18.5	880	100	140	100	190	33	5.2	ND	73	ND	840	1.1	-31.1	0.9	-23.7	-8.3	100	0.00077	1.9.E+08	1.3.E+08	3.9.E+06	2.1.E+05
20-24 cm	22	680	100	140	100	200	11	11	ND	91	ND	680	1.8	-30.6	1.1	-20.7		81	0.00072	1.7.E+08	2.5.E+08	2.5.E+07	4.5.E+06

*Not detected
