# Supplementary material for: Vertical distribution of methanotrophic archaea in an iron-rich groundwater discharge zone
Source: PLoS One. 2025 Feb 24;20(2):e0319069. doi: 10.1371/journal.pone.0319069 (PMC11849818; doi:10.1371/journal.pone.0319069)
Supplement: S3 Table — (PDF) [file pone.0319069.s003.pdf]

S3 Table. Summary of the 16S rRNA gene amplicon libraries.

Depth (cmbsf)	Number of sequence read	Observed features	Shannon index	Pielou's evenness	Faith's PD
0-1 cm	52,116	797	6.87	0.71	65.6
1-2 cm	86,252	1,605	8.99	0.84	112.0
2-3 cm	79,595	1,651	9.23	0.86	108.5
3-4 cm	61,629	1,312	8.90	0.86	93.0
4-5 cm	65,291	1,283	8.71	0.84	92.9
5-7 cm	70,236	1,237	8.39	0.82	89.0
7-9 cm	72,593	996	7.90	0.79	74.0
9-11 cm	72,543	865	7.48	0.77	65.0
11-14 cm	79,499	926	7.44	0.75	67.2
14-17 cm	74,119	684	6.31	0.67	51.7
17-20 cm	91,439	585	5.78	0.63	46.5
20-24 cm	72,404	697	6.30	0.67	53.6
