# Supplementary figures and images for: Vertical distribution of methanotrophic archaea in an iron-rich groundwater discharge zone
Source: PLoS One. 2025 Feb 24;20(2):e0319069. doi: 10.1371/journal.pone.0319069 (PMC11849818; doi:10.1371/journal.pone.0319069)

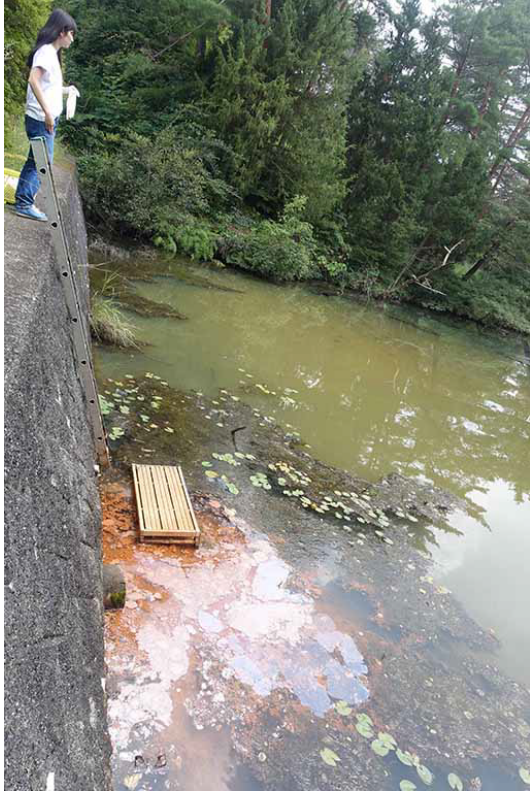

**S1 Fig.** Location of the sampling sites. Overview of the groundwater discharge point of Budo Pond.

Supplement: S1 Fig — Overview of the groundwater discharge point of Budo Pond. (PDF) [file pone.0319069.s004.pdf]

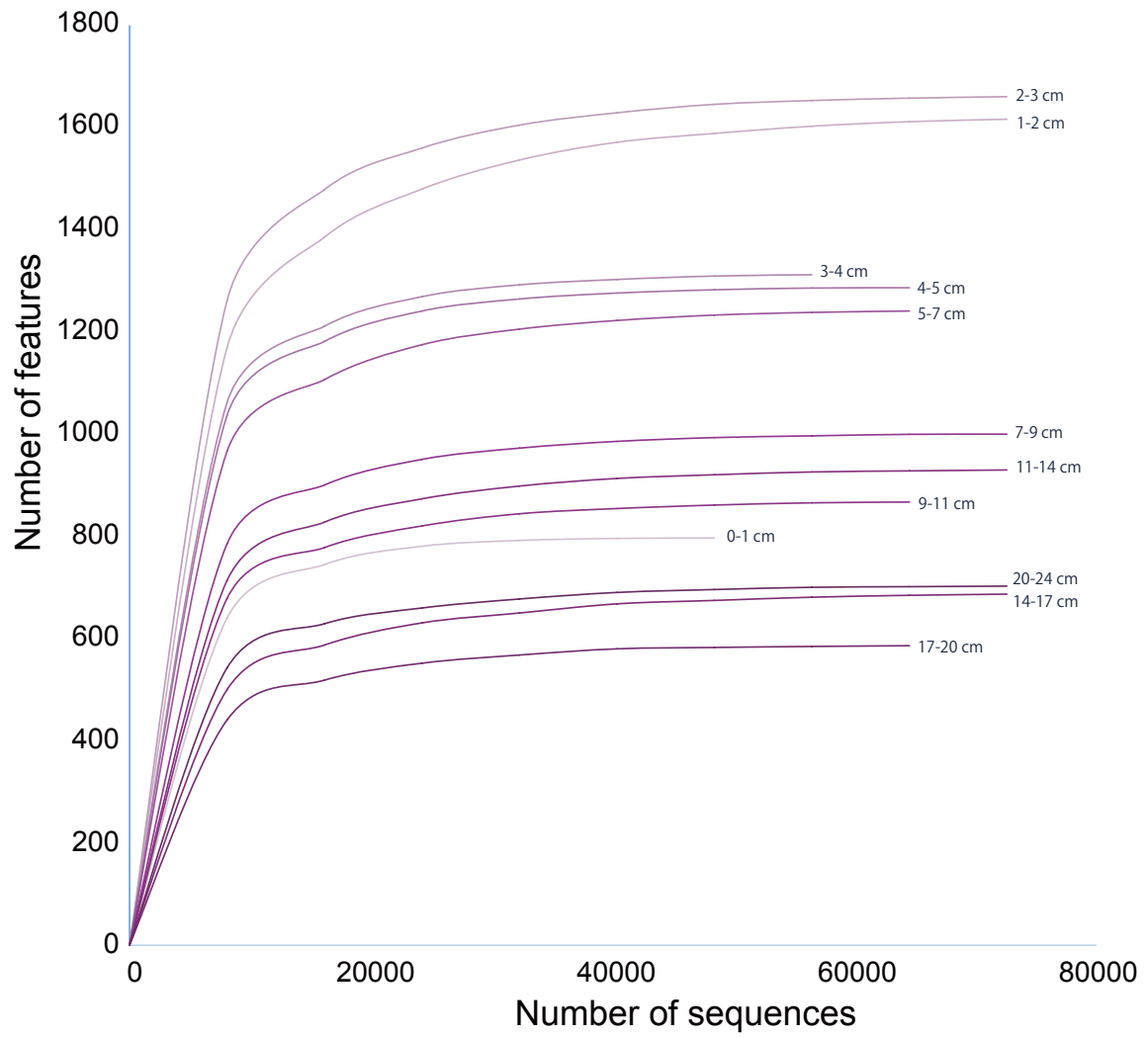

**S3 Fig.** Rarefaction curves for the 16S rRNA gene amplicon libraries of the sediment.

Supplement: S3 Fig — (PDF) [file pone.0319069.s006.pdf]
