# Supplementary material for: Vertical distribution of methanotrophic archaea in an iron-rich groundwater discharge zone
Source: PLoS One. 2025 Feb 24;20(2):e0319069. doi: 10.1371/journal.pone.0319069 (PMC11849818; doi:10.1371/journal.pone.0319069)
Supplement: S2 Fig — The red-shaded layers represent the possible depth ranges of active Fe-AOM, as determined in Fig 1. (PDF) [file pone.0319069.s005.pdf]

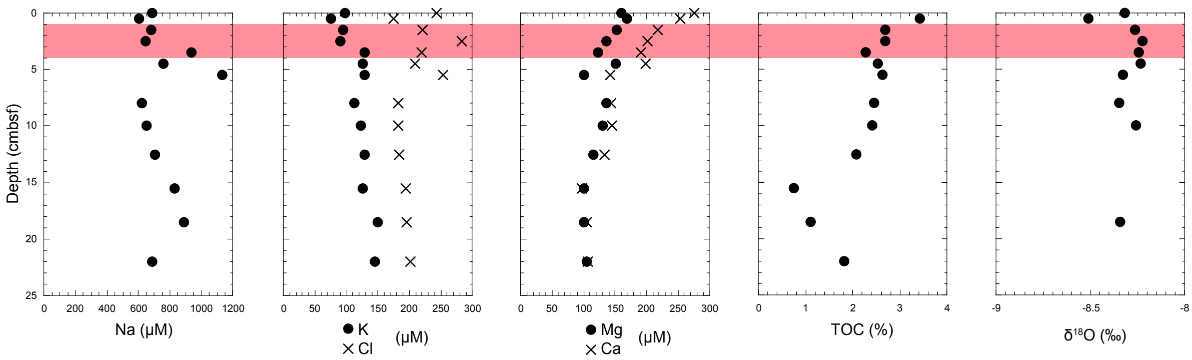

S2 Fig. Depth profiles of pore water Na, K, Cl, Mg, Ca, TOC, and the oxygen isotopic composition of water. The red-shaded layers represent the possible depth ranges of active Fe-AOM, as determined in Fig. 1.
