# Supplementary material for: Vertical distribution of methanotrophic archaea in an iron-rich groundwater discharge zone
Source: PLoS One. 2025 Feb 24;20(2):e0319069. doi: 10.1371/journal.pone.0319069 (PMC11849818; doi:10.1371/journal.pone.0319069)
Supplement: S4 Fig — The two principal coordinate axes explain 75.83% of variation. (PDF) [file pone.0319069.s007.pdf]

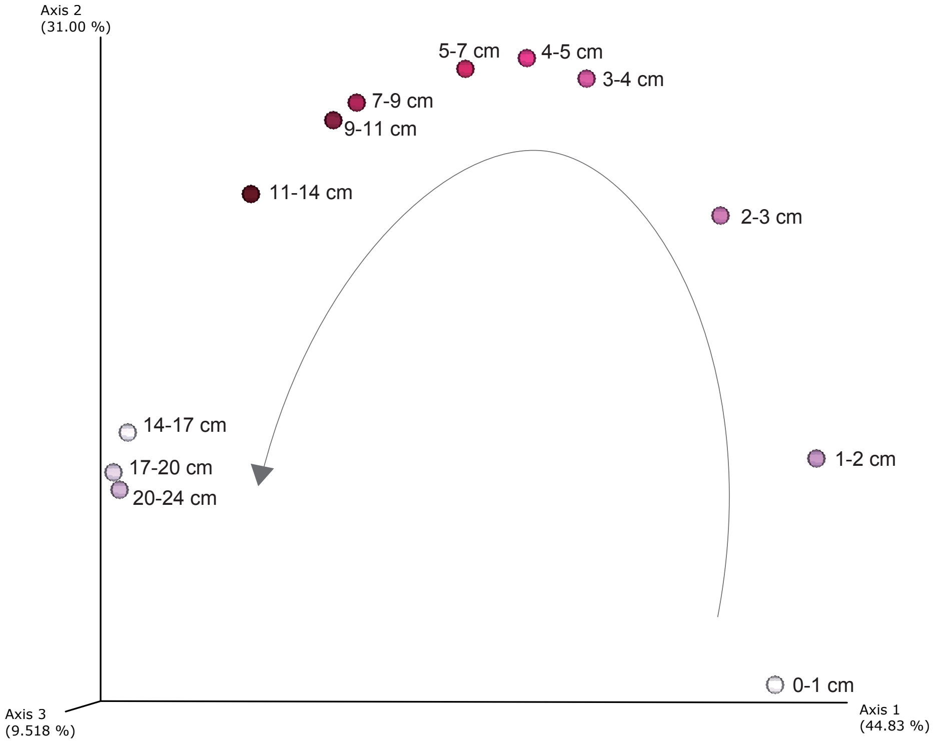

S4 Fig. Beta-diversity visualized using principal coordinate analysis (PCoA). The two principal coordinate axes explain 75.83% of variation.
