# Supplementary material for: Vertical distribution of methanotrophic archaea in an iron-rich groundwater discharge zone
Source: PLoS One. 2025 Feb 24;20(2):e0319069. doi: 10.1371/journal.pone.0319069 (PMC11849818; doi:10.1371/journal.pone.0319069)
Supplement: S5 Fig — Sediment samples at depths of 1.5 cm (A) and 4.5 cm (B) were selected as examples to show cell morphology. (PDF) [file pone.0319069.s008.pdf]

(A)

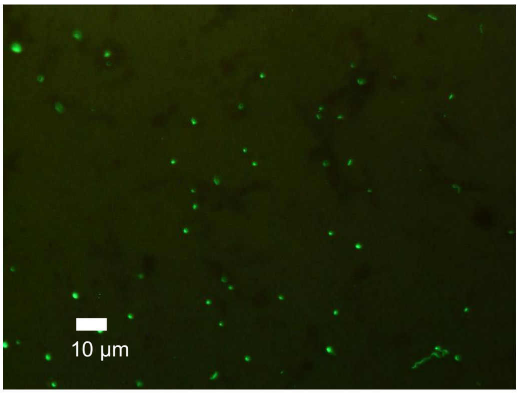

(B)

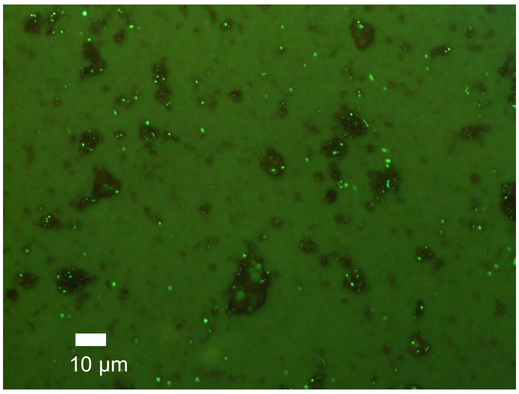

S5 Fig. Fluorescence microscopic image of microbial cells from the Budo Pond sediment. Sediment samples at depths of 1.5 cm (A) and 4.5 cm (B) were selected as examples to show cell morphology. Cells were stained with SYBR Green I. The bar indicates 10 µm.
